# Socio-demographic characteristics and associated factors influencing cervical cancer screening among women attending in St. Paul’s Teaching and Referral Hospital, Ethiopia

**DOI:** 10.1186/s12905-020-00927-5

**Published:** 2020-04-06

**Authors:** Abebe Belete Woldetsadik, Abebe Feyissa Amhare, Sintayehu Tsegaye Bitew, Leilei Pei, Jian Lei, Jing Han

**Affiliations:** 1 School of Public Health, Xi’an Jiaotong University, Health Science Center, Xi’an, 710061 Shaanxi China; 2 Shenzhen Institute, Xi’an Jiaotong University, Shenzhen, Guangzhou 518057 People’s Republic of China

**Keywords:** Cervical cancer, Factors, Screening, Sociodemographic characteristics, Women

## Abstract

**Background:**

In Ethiopia, cervical cancer is the second most frequent cancer among women aged 15 to 44 years old. Cervical cancer screening is an effective measure to enhance the early detection of cervical cancer for prevention. However, the magnitude of cervical cancer screening is less than 1%. This study aimed to determine the influence of sociodemographic characteristics and related factors on screening.

**Method:**

A hospital-based cross-sectional study has been conducted from July to September 2017. Data have been collected using interviewer-administered questioner among 425 women (18–49 years age) who visited the family health department at St. Paul’s Hospital. Descriptive statistics, chi-square, univariate and multivariate logistic regression were used for data analysis.

**Result:**

Of the 425 study participants, only 12.2% of women have been screened within the past 3 years. Women in the age range of 40–49 years old were more likely to be screened (36.1%) than women age 18–29 years (8%). Women living in urban were more likely to be screened (15.9%) than women living in rural (3.9%). Other factors including low monthly income, unlikely chance of having cancer, lack of knowledge, and fear test outcome were significantly associated with the low uptake of screening.

**Conclusion:**

This study revealed that the uptake of cervical cancer screening was low. Women in the potential target population of cervical cancer screening were just a proportion of all studied age groups and screening in them was more common than in younger women. Besides, rural residence, low monthly income, and lack of knowledge were important predictors for low utilization of cervical cancer screening practice.

## Background

Cervical cancer is one of the most public health burdens in the world [1, 2]. Globally, more than 569,000 new cervical cancer and 311,000 women death by cervical cancer have been reported annually by the International Agency for Research on Cancer (IARC) Global Cancer Observatory [2, 3]. In Africa, more than 119,000 new cervical cases and 81,000 deaths of cervical cancer have been reported annually by IARC Global Cancer Observatory [2]. Over the past 30 years, the incidence and mortality rate of cervical cancer has been increased every year by 0.6 and 0.46% respectively [4]. In Sub-Saharan Africa countries, cervical cancer comprises 20 to 25% of total cancer cases, which is two times that of women in the world [5]. World Health Organization (WHO) has reported that low and middle-income countries are taking the highest burden of cervical cancer. This is mainly due to lack of effective screening schedules and limited uptake of pelvic examination [6].

In Ethiopia, over 27.19 million females who are 15 years and above are at risk to develop cervical cancer [7]. The recent study estimated that there are annually 6294 new cases and 4884 cervical cancer deaths in Ethiopia [2]. It is the second most frequent cancer among women 15 to 44 years old. Despite this fact, less than 1% of women underwent cervical cancer screening every 3 years [7, 8]. Ethiopia has no separate cervical cancer prevention and control program; it has been done together with the reproductive care system until recent years [9]. In 2009, the Federal Ministry of Health (FMOH) collaboration with Pathfinder International had established a pilot project to provide the service in 14 health institutions in different regions of the country. Since 2015, FMOH has launched a new directive guideline to scale up cervical cancer prevention and control program by health workers and concerned stockholders to implement the program; Visual Inspection Acetic acid (VIA) and cytology test were used as a national cervical cancer screening approach [10].

The previous study also reported that Human Papilloma Virus (HPV) infection, multiple sexual partners, smoking, high parity, long-term hormonal contraceptive use, and co-infection with Human Immune deficiency Virus (HIV) as risk factors of cervical cancer [11].

Cervical cancer progression depends on the stage of a patient at first contact at the health facility. In developing countries including Ethiopia, most patients are coming to the health facility after the advanced stage of the disease [12, 13]. Therefore, the improvement is becoming reduced even using multiple treatment modalities, such as surgery, radiology or chemotherapy. Several reasons are noted in different studies; for instance, location, access to the health facility, educational level, financial capability, and later visit of their doctor [13, 14].

Effective screening program reduces the incidence and mortality of cervical cancer as the disease is potentially preventable [15–17]. In developed countries, routine cervical cancer screening prevents up to 80% through early detection and management of pre-cancerous cervical lesions [18–20]. Whereas in developing countries, the proportion of pelvic examination was very low (< 1% in Ethiopia to 23% in South-Africa) [18]. These differences are due to different factors like financial constraints, complex structural and cultural issues [21]. Women have differed in cervical screening even with a similar opportunity in Ethiopia. Besides, there is no plenty of literature related to this topic found in Ethiopia. Therefore, the purpose of this study is to determine the influence of socio-demographic characteristics and related factors on cervical screening among women who attended St. Paul’s teaching and referral hospital, Ethiopia.

## Methods

### Study design and study area

The hospital-based cross-sectional study design has been conducted from July to September 2017 at the family health department of St. Paul’s Teaching and Referral Hospital (SPTRH). SPTRH is one of the specialized referral hospitals located in Addis Ababa, the capital city of Ethiopia. This hospital has been serving over five million catchment population living in urban, semi-urban and rural areas.

### Sample size determination and sampling

Based on available hospital registration records, approximately about 16,800 women aged 18–49 years visit the outpatient clinic provide service at the family health department per year.

The required sample size was estimated using a single population proportion formula with 95% CI, 5% margin of error, and 50% of population proportion was assumed as the prevalence of screening to maximize sample size because the prevalence of previous studies was very small. By adding a 10% non-response rate, the total calculated sample size was 425. Since the study group of the population was dynamic, purposive sampling method was used to select 425 women based on arrival record book using inclusion criteria (women aged ≥18 and less than or equal to 49 years, voluntariness, and who visited outpatient clinic during the study period) and exclusion criteria (women who had hysterectomy and seriously ill) were selected until the required sample was achieved.

### Data collection technique

Quantitative data were collected using an interviewer-administered questionnaire concerning cervical cancer and screening. Cervical cancer screening within the last 3 years was considered as the outcome variable (1 for the screen, and 0 for unscreened). A predictive variable of interest includes socio-demographic characteristics (age, income, occupation, residence, parity, religion, marital status, and educational level), knowledge, and perception towards cervical cancer and its screening, exposure status for the risk factor, and barriers concerning cervical cancer screening. The questionnaire was adopted from a standardized questionnaire [22] and contextualized based on the study objective. Initially, the questionnaire was developed in English and translated into local language, Amharic by language experts. A pilot study had been carried out among 22 participants before actual study, and an amendment was done accordingly. Trained nurses had collected the data using face to face interview.

### Data analysis

Data were double entered into Epi-data version 3.0 and transported to SPSS version 24.0 for analysis. Descriptive information was presented using tables and figure. Frequency and percentage were performed to compute the proportion of groups among the study participants. Chi-square analysis was used to determine the association between predictors and outcome variables. Univariate and multivariate logistic regression were used and a *P* value of less than 0.2 in univariate analysis was entered to multivariate logistic regression analysis to adjust the effect of confounders to the outcome variable. Odds ratios with 95% Confidence Intervals were computed; a *P* value < 0.05 was considered as statistically significant.

## Results

### Sociodemographic characteristics of the study participants

A total of 425 respondents (response rate 100%) had participated in this study. Of 425 respondents, 202 (47.5%) participants were 30–39 years age, 333 (78.4%) were married, and 169 (39.8%) were housewives. More than one-quarter of respondents (29.6%) were attended primary education and 55.3% had one or two births. More than 30 % (30.4%) of participants were residing in the rural area and 20.1% had no access to screening in a nearby health facility. More than half of respondents (55.2%) had monthly income less than 2000 Ethiopia Birr (75 USA dollars) (Table 1).

**Table 1:** Socio-demographic characteristics of respondents (*n* = 425)

Characteristics	Frequency (*n*)	Percent (%)
**Age in year**		
18–29	187	44.0
30–39	202	47.5
40–49	36	8.5
**Marital status**		
Single	75	17.6
Married	333	78.4
Divorce/ Windowed	17	4.0
**Religion**		
Orthodox	272	64.0
Muslim	85	20.0
Protestant	61	14.4
Other	7	1.6
**Educational status**		
Primary & below	126	29.6
Secondary & higher	299	70.4
**Occupational status**		
Governmental employee	94	22.1
Self-employed	162	38.1
Housewife	169	39.8
**Parity**		
Nulliparous	134	31.5
1–2 birth	235	55.3
3 & above	56	13.2
**Residence**		
Urban	296	69.6
Rural	129	30.4
**Monthly income**		
< 2000 Birr	235	55.3
2000–3999 Birr	105	24.7
4000^+^	85	20.0

### Association between socio-demographic characteristics and cervical cancer screening

**Fig. 1:**
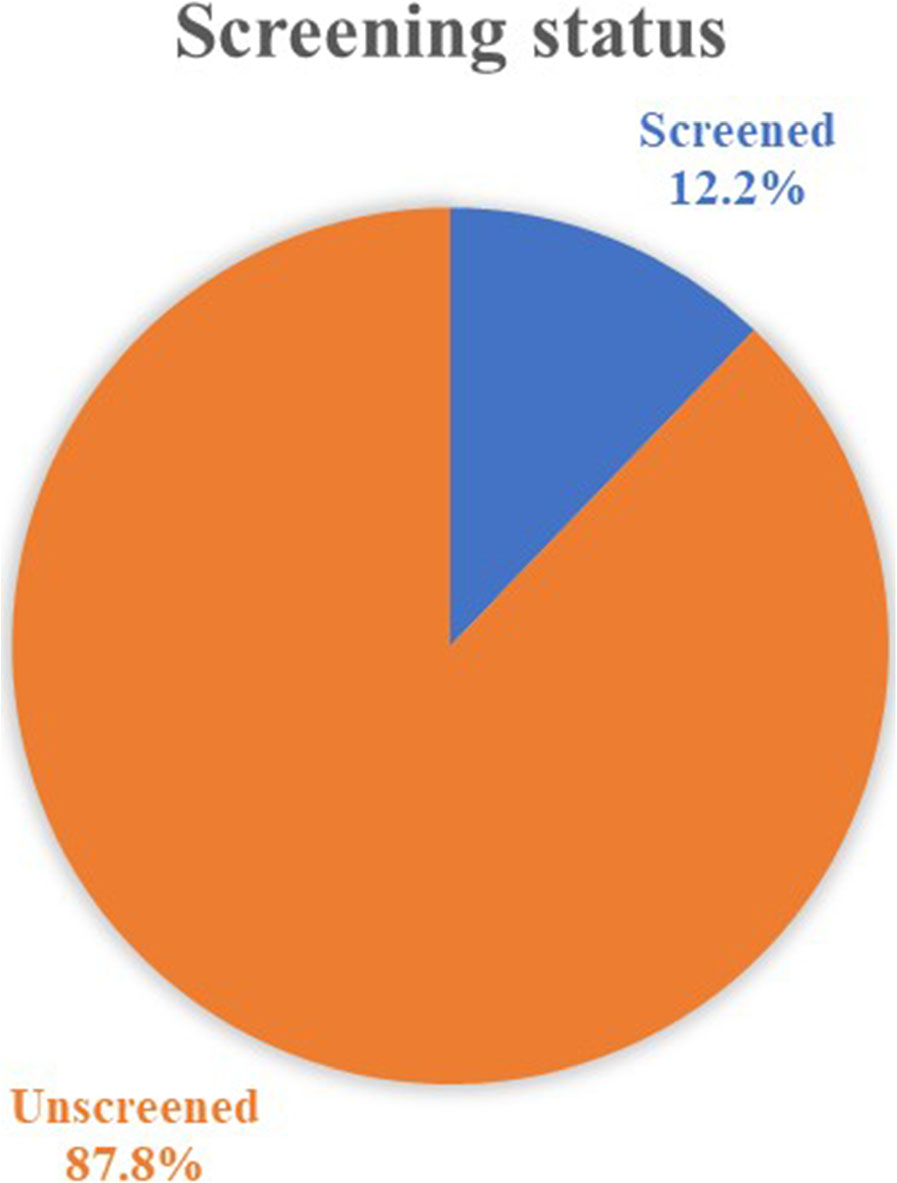
Cervical screening status: Cervical cancer screening practice of participants during last 3 years

Among 425 study participants, only 12.2% of women have been screened within the past 3 years as illustrated in Fig. 1. The proportion of women who are screened at the age of 18–29 was 8% which was lower than women aged 30–39 (11.9%) and 40–49 (36.1%). Logistic regression analysis identified that, geographically, women who live in rural area were less likely to be screened [OR = 0.30, 95% CI: (0.11–0.85)] than women who live in urban area. Regarding respondents’ age, women in the 40–49 years age group were more likely to undergo cervical cancer screening practice [OR = 3.58, 95% CI: (1.21–10.58)] compared with women aged 18–29 years old. Additionally, the respondents’ screening was differed by occupation, self-employed women were more likely to be screened [OR = 2.58, 95% CI: (1.06–6.27)] than governmental employed women. Compared with nulliparous women, multiparous women more likely participated in cervical cancer screening [OR = 2.79, 95% CI: (1.05–7.39)]. Regarding income of respondents, women who had been earned 3000–3999 Birr (111–148 USA dollar) per month [OR = 2.93, 95% CI: (1.32–6.51)] and those who had earned greater than 4000 Birr (148 USA dollar) [OR = 2.97, 95% CI: (1.27–6.92)] were more likely seeking for screening than women who had been earned less than 2000 Birr (75 USA dollar) per month. However, marital status and educational level of respondents didn’t show significant association (*P* > 0.05) as shown in Table 2.

**Table 2:** Socio-demographic characteristics and participation in cervical screening during last 3 years: Logistic regression

Characteristics	Unscreened n (%)	Screened n (%)	Odds ratios	
			Crude (95% CI)	Adjusted (95% CI)
**Age**				
18–29 year	172 (92.0)	15 (8.0)	1.00	1.00
30–39 year	178 (88.1)	24 (11.9)	1.55 (0.79–3.05)	1.05 (0.48–2.30)
40–49 year	23 (63.9)	13 (36.1)	6.48 (2.74–15.33)	3.58 (1.21–10.58)^*^
**Marital status**				
Single	70 (93.3)	5 (6.7)	1.00	1.00
Married	291 (87.4)	42 (12.6)	2.02 (0.77–5.29)	1.44 (0.46–4.55)
Divorce or Windowed	12 (70.6)	5 (29.4)	5.83 (1.46–23.25)	1.52 (0.27–8.46)
**Educational level**				
Primary and below level	121 (96.0)	5 (4.0)	1.00	1.00
Secondary and above	252 (84.3)	47 (15.7)	4.51 (1.75–11.64)	2.71 (0.94–7.79)
**Occupation**				
Governmental	85 (90.4)	9 (9.6)	1.00	1.00
Self-employed	135 (83.3)	27 (16.7)	1.89 (0.85–4.21)	2.58 (1.06–6.27)^*^
Housewife	153 (90.5)	16 (9.5)	0.99 (0.42–2.33)	1.45 (0.56–3.78)
**Parity**				
Nulliparous	126 (94.0)	8 (6.0)	1.00	1.00
1–2 birth	202 (86.0)	33 (14.0)	2.57 (1.15–5.75)	2.79 (1.05–7.39)^*^
3 and above	45 (80.4)	11 (19.6)	3.85 (1.46–10.18)	3.31 (0.94–11.59)
**Monthly income**				
< 2000 Birr	221 (94.0)	14 (6.0)	1.00	1.00
2000–3999 Birr	85 (81.0)	20 (19.0)	3.71 (1.79–7.69)	2.93 (1.32–6.51)^*^
4000 and above Birr	67 (78.8)	18 (21.2)	4.24 (2.00–8.98)	2.97 (1.27–6.92)^*^
**Residence**				
Urban	249 (84.1)	47 (15.9)	1.00	1.00
Rural	124 (96.1)	5 (3.9)	0.21 (0.08–0.55)	0.30 (0.11–0.85)^*^

^*^statistically significant at *P* value < 0.05

### Risk factors and cervical screening practice

Study participants who exposed to cervical cancer risks had low participation in cervical screening. Table 3 indicates that the risk factor and cervical screening practice of participants. According to this study, among screened women, 23.1% had a history of sexual contact before 18 years old. Besides, 57.7% of screened women didn’t use the condom during sexual contact. Also, 28.8% of screened women had a history of either hypertension, diabetes, infertility or irregular menses cases.

**Table 3:** Risk factors and cervical screening practice

Risk factors	Screening status	
	Unscreened n (%)	Screened n (%)
**Age at first sex, < 18-year-old**		
No	274 (73.5)	40 (76.9)
Yes	99 (26.5)	12 (23.1)
**Multiple sexual partners**		
No	332 (89.0)	40 (76.9)
Yes	41 (11.0)	12 (23.1)
**Frequency of condom use**		
Not use	260 (69.7)	30 (57.7)
Occasionally	87 (23.3)	15 (28.8)
Always	26 (7.0)	7 (13.5)
**History of smoking**		
No	363 (97.3)	51 (98.1)
Yes	10 (2.7)	1 (1.9)
**COC pills use**		
No	161 (43.2)	23 (44.2)
< 1 year	95 (25.5)	7 (13.5)
> 1 year	117 (31.4)	22 (42.3)
**History of STD**		
No	348 (93.3)	45 (86.5)
Yes	25 (6.7)	7 (13.5
**HIV test**		
No	25 (6.7)	0 (0.0)
Yes	348 (93.3)	52 (100.0)
**HIV status (***N* **= 400)**		
Negative	333 (89.3)	47 (95.7)
Positive	15 (4.0)	5 (4.3)
**History of HPT, DM, IF or IM**		
No	296 (79.4)	37 (71.2)
Yes	77 (20.6)	15 (28.8)

*COC* Combined oral contraceptive, *STD* Sexually transmitted disease, *HPT* Hypertension, *DM* Diabetic mellitus, *IF* Infertility, *IM* Irregular menses

### Knowledge and perception factors associated with cervical screening during the last 3 years

Regarding knowledge of cervical cancer screening, women who heard cervical cancer screening benefits were more likely to be screened [OR = 4.11, 95% CI: (1.12–15.04)] than those who had not. Respondents who knew about precancerous cervical dysplasia which could happen without symptoms were more likely to be screened [OR = 2.09, 95% CI: (1.06–4.10)] than women who didn’t know.

Women who agreed by the thought that “I can’t do screening even though beneficial” were less likely to be screened [OR = 0.10, 95% CI: (0.02–0.43)] as compared with counterparts. Women who likely felt the possibility of getting cervical cancer were more likely to be screened than counterparts [OR = 5.36, 95% CI: (2.12–13.55)]. Also, those who perceived the chance of getting cervical cancer were more likely to be screened [OR = 3.40, 95% CI: (1.51–7.69)] as compared with women who unlikely perceived (Table 4).

**Table 4:** Knowledge and perception factors associated with cervical screening during the last 3 years

Characteristics	Unscreened n (%)	Screened *n* (%)	Odds ratios	
			Crude (95% CI)	Adjusted (95% CI)
**Heard about cervical cancer**				
No	151 (40.5)	7 (13.5)	1.00	1.00
Yes	222 (59.5)	45 (86.5)	4.37 (1.92–9.96)	0.59 (0.16–2.18)
**Heard about CC screening**				
No	179 (48.0)	6 (11.5)	1.00	1.00
Yes	194 (52.0)	46 (88.5)	7.07 (2.95–16.96)	4.11 (1.12–15.04)^*^
**PCD can happen without symptom**				
No	262 (70.2)	21 (40.4)	1.00	1.00
Yes	111 (29.8)	31 (59.6)	3.48 (1.92–6.33)	2.07 (1.06–4.10)^*^
**CC is killer, if undetected and untreated early**				
Disagree	36 (9.7)	2 (3.8)	1.00	1.00
Agree	226 (60.6	44 (84.6)	3.50 (0.81–15.09)	3.64 (0.75–17.59)
I don’t know	111 (29.8)	6 (11.5)	0.97 (0.19–5.04)	1.27 (0.205–7.85)
**Overall Knowledge about cervical cancer**				
Not knowledgeable	298 (79.9)	21 (40.4)	1.00	1.00
Knowledgeable	75 (20.1)	31 (59.6)	5.87 (3.19–10.79)	2.48 (1.18–5.19)^*^
**Like many women, I am susceptible to CC.**				
No	161 (43.2)	10 (19.2)	1.00	1.00
Yes	212 (56.8)	42 (80.8)	3.19 (1.55–6.55)	1.36 (0.57–3.26)
**Even if I see screening is beneficial, I can’t do so.**				
Disagree	254 (68.1)	43 (82.7)	1.00	1.00
Agree	104 (27.9)	2 (3.8)	0.11 (0.03–0.48)	0.10 (0.02–0.43)^*^
Indifferent	15 (4.0)	7 (13.5)	2.76 (1.06–7.15)	3.87 (1.16–12.86)^*^
**Chance of getting CC.**				
Unlikely	278 (74.5)	17 (74.5)	1.00	1.00
Somewhat	63 (16.9)	20 (16.9	5.19 (2.57–10.48)	3.41 (1.51–7.69)^*^
Likely	32 (8.6)	15 (8.6)	7.67 (3.49–16.80)	5.36 (2.12–13.55)^**^

*CC* Cervical Cancer, *PCD* Pre-malignant cervical cancer dysplasia

^**^Statistically significant at *P* value < 0.001, ^*^Significant at *P* value < 0.05

### Barrier factors and participation in cervical screening during the last 3 years

Women who fear the test outcome of cervical cancer were less likely to be screened [OR = 0.50, 95% CI: (0.23–0.92)] than women who didn’t fear. Based on cervical screening information, those who had its information more likely to be screened [OR = 2.01, 95% CI: (1.03–3.95] as compared with women who need more information. Respondents who knew the place of cervical screening services were more likely to be screened [OR = 11.83, 95% CI: (3.54–39.51)] than those who didn’t know. Furthermore, women who had a history of cervical illness were more likely to be screened [OR = 8.64, 95% CI: (2.36–31.63)] as compared with women who felt healthiness (Table 5).

**Table 5:** Association between barrier factors and cervical screening (*n* = 425)

Barrier factors	Unscreened n (%)	Screened n (%)	Odds ratios	
			Crude (95% CI)	Adjusted (95% CI)
**Fear test outcomes**				
No	221 (59.2)	40 (76.9)	1.00	1.00
Yes	152 (40.8)	12 (23.1)	0.44 (0.22–0.86)	0.47 (0.23–0.92)^*^
**Is a test too embarrassing**				
No	313 (83.9)	44 (84.6)	1.05 (0.47–2.35)	2.31 (0.93–5.75)
Yes	60 (16.1)	8 (15.4)	1.00	1.00
**Need more information**				
No	141 (37.8)	24 (46.2)	1.41 (0.79–2.53)	2.01 (1.03–3.95)^*^
Yes	232 (62.2)	28 (53.8)	1.00	1.00
**Difficult to talk to a provider**				
No	321 (86.1)	41 (78.8)	0.60 (0.29–1.25)	0.61 (0.27–1.42)
Yes	52 (13.9)	11 (21.2)	1.00	1.00
**Don’t know where to get the service**				
No	227 (60.9)	49 (94.2)	10.51 (3.22–34.33)	11.83 (3.54–39.51)^**^
Yes	146 (39.1)	3 (5.8)	1.00	1.00
**Clinic far from home**				
No	290 (77.7)	46 (88.5)	2.19 (0.91–5.32)	2.17 (0.81–5.81)
Yes	83 (22.3)	6 (11.5)	1.00	1.00
**Perceived poor quality of service**				
No	305 (81.8)	37 (71.2)	0.55 (0.29–1.06)	0.60 (0.28–1.27)
Yes	68 (18.2)	15 (28.8)	1.00	1.00
**I had never Hx of cervical illness**				
No	292 (78.3)	49 (94.2)	4.53 (1.38–14.91)	8.64 (2.36–31.63)^*^
Yes	81 (21.7)	3 (5.8)	1.00	1.00

*Hx* History

^**^Statistically significant at *P* value < 0.001, ^*^Significant at *P* value < 0.05

## Discussions

Cervical cancer screening is the easiest, simple and easily affordable choice to prevent and reduce maternal mortality associated with cervical cancer. This study found that only 12.2% of women had a practice of cervical cancer screening; however, this finding is higher than the national data reported by the Institute Catalan of Oncology (ICO) in 2017 which showed that cervical screening practices in Ethiopia were less than 1% [8]^.^ The differences might be due to the countrywide report, poor registration system or the study setup. But, it is in line with the finding of a study in the Southern part of Ethiopia (11.5%) [23]. However, it is lower than the finding of a study done in the Northern part of Ethiopia (19.8%) [22]. This might be due to the difference in the study setup, or the former study was conducted at the place where one of the pilot projects allocated areas in the country. This made better screening access for women participated in the former study. Similarly, the present finding is also significantly lower than the finding of Portland Jamaica study (66%) [6]. This variability might be due to inadequate access to health facility, screening tools, skilled professionals, better governmental concern to cervical cancer prevention and control, and availability of Pap test at the health institution.

Our study found that age was significantly associated with the uptake of cervical cancer screening. The study revealed that women in the youngest age (18–29 years) were less likely to be screened compared with women in the older age (40–49 years). The finding was similar to studies done in South Africa and Portland Jamaica [6, 20]. Older women have a higher likelihood to develop cervical cancer than younger, however, it is important to consider as they are sexually active.

Rural residence was significantly associated with low uptake of screening in this study. Related studies were done in Nepal [19] and Uganda [24] showed that rural women were less likely to receive screening. Another study conducted in Mexico revealed that women who reside in rural areas had a significantly greater geographic accessibility burden when compared to non-rural areas (4.4 km vs 2.5 km and 4.9 min vs 3.0 min) for screening due to these rural women were less likely underwent to cervical screening [25]. In Ethiopia, most screening facilities were available in urban hospitals while 80% of the population lives in rural areas; on the other hand, those hospitals were overburdened by advanced cases/chronic diseases; because of this reason, the participation in screening is compromised. Therefore, it needs to address the rural population by decentralizing the service to rural health centers.

This study found that nulliparous women were associated with the low uptake of cervical cancer screening, which means nulliparous women were less likely screened than multiparous women. This finding is consistent with a study conducted in Thailand that revealed women who had no children had less practice of cervical screening [26]. Underuse of screening nulliparous women is not a significant public health concern while they are less likely a chance to develop the disease compared to multiparous women. However, it is important to engage those women in prevention activities as all women are at risk of cervical cancer.

Occupational status significantly influences the uptake of cervical cancer screening in this study; which means self-employed women were more likely to be screened compared with governmental employed women. This finding concurs with a study conducted in Northern Greece which reported that women with full-time employees had higher compliance to participate in cervical screening [27]. This is might be due to those government workers have no time to go or maybe due to negligence, therefore it is better to address effective intervention including cervical cancer awareness and preventive measures.

In this study, women who had low monthly income were significantly associated with low uptake of cervical cancer screening. This finding is argued with a study done in Ontario that showed women with low monthly income were less likely engaging in cervical screening [28]. Also, a study conducted in Belgrade reported that lower economic status has a great impact on cervical screening even fee-free available services [29]. In fact, in Ethiopia, cervical screening service is freely available but different reasons are identified as limitations to use the service among low-income women; for instance, transportation fees, childcare cost, and frequent attending to the subsequent follow-up visit.

Lack of information towards cervical screening benefits could influence the uptake of screening. This study found that respondents who had no information about cervical cancer screening benefit less participated in cervical screening. A related study from Omaha Nebraska stated that the majority of women in the study never heard about cervical cancer and they had less practice of pap test [30]. Therefore, providing relevant information on screening and its benefit from concerned stakeholders is crucial to ensure cervical cancer screening.

This study also found a significant association between knowledge of premalignant dysplasia and uptake of cervical screening. Our study showed that the majority of respondents didn’t have knowledge of the occurrence of pre-malignant dysplasia without signs or symptoms, in contrast, women who knew about the pre-cancerous condition of cervical cancer were more likely to be screened compared with women who didn’t. A study from Kisumu Kenya showed that 63.2% of the respondents had no knowledge regarding signs and symptoms of cervical cancer and had less practice of cervical cancer screening [31].

There were strong associations between self-perceived to get cervical cancer and uptake of screening in this survey; which means women who perceived unlikely to get cervical cancer had been less likely screened. A qualitative study conducted in the British showed that women who felt the low perceived risk of cancer were less likely to be screened [32]. Additional studies from Vietnamese Americans [33] and qualitative study from Kurdish described that women who believed as a lower risk group of getting cancer were less willing to perform the test [34]. This perception might be related to religion or belief in destiny; therefore, the participation of religious leaders in controlling the cervical cancer program is better.

Moreover, the current study showed that the test result was a significant barrier to the uptake of cervical cancer screening; which means women who fear test outcome cervical cancer had less practice of cervical screening. Besides, barrier factors such as lack of information, unknown place of service, and felt wellness were shows significant association with low screening uptake. These findings have also been documented in other studies [22, 24, 33] Therefore, it needs to address these barriers to increase the uptake of screening practice.

This study has some limitations. It is a hospital-based study that is difficult to represent the entire population. It has a small sample size that can reduce statistical power and generalization. Other limitations of this study are recall bias and selection bias. As the sampling method of this study was purposive, it might be led to selection bias and decrease reliability. Lack of RR analysis methods also another limitation of this study as RRs would fit with common outcomes rather than OR in which some of the point estimates are not easy to interpret. The cross-sectional design investigates prevalence and associations rather than causality. Thus, future research needs to replicate these findings using prospective studies/longitudinal study design. A high percentage of the participation rate is considered the strength of this study.

## Conclusion

This study aimed to determine the influence of sociodemographic characteristics and related factors for the low uptake of cervical cancer screening among women. Based on our study findings, women in the potential target population of cervical cancer screening were just a proportion of all studied age groups and screening in them was more common than in younger women. In addition, rural residence, low monthly income, governmental employee, lack of knowledge and wrong perception towards cervical cancer were significantly associated with the low uptake of cervical cancer screening. Therefore, we suggest that improving the health care system by giving due attention to the rural residence, low-income population, awareness creation towards screening, and associated risk factors including delivering the service to the government employee in their workplace. Furthermore, our study contributes insightful basis to develop strategies to prevent and control cervical cancer based on the sociodemographic profile.

## Data Availability

Data are available from abwolde69@gmail.com on a reasonable request.

## Abbreviations

CC: Cervical cancer
COC: Combined oral contraceptive
DM: Diabetes mellitus
FMOH: Federal Ministry of Health
HIV: Human Immune deficiency Virus
HPV: Human Papilloma Virus
IARC: International Agency for Research on Cancer
ICO: Institute Catalan of Oncology
IF: Infertility
IM: Irregular menses
STRH: St. Paul’s Teaching and Referral Hospital
VIA: Visual Inspection Acetic acid
WHO: World Health Organization
